# Current Concepts in Scapholunate Instability Without Arthritic Changes

**DOI:** 10.1007/s43465-023-00839-0

**Published:** 2023-03-07

**Authors:** Melanie Amarasooriya, Terrence Jose Jerome, Lisa Tourret

**Affiliations:** 1 Department of Orthopedic and Trauma Surgery, Flinders Medical Centre and Flinders University, Bedford Park, South Australia 5042 Australia; 2 Orthopedic Surgeon, Ministry of Health, Colombo, Sri Lanka; 3 Hand and Reconstructive Microsurgery, Olympia Hospital and Research Centre , Trichy, India; 4 Hand and Upper Limb Surgeon, Brighton and Sussex University Hospitals, NHS Trust, Brighton, UK

**Keywords:** Scapholunate, Wrist instability, Scapholunate ligament, Instability, SLAC, DISI

## Abstract

Scapholunate instability (SLI) is the most common carpal instability described. SLI leads to a degenerative arthritic pattern known as scapholunate advanced collapse (SLAC). Diagnosis of SLI can be challenging in pre-dynamic and dynamic stages. CT arthrogram, MR arthrogram and dynamic fluoroscopy are helpful in diagnosis while arthroscopy remains the gold standard. SLI is a multi-ligament injury, which involves not only the scapholunate interosseous ligament (SLIL) but also the extrinsic carpal ligaments. Hence, it is better described as an injury compromising the ‘dorsal scapholunate(dSLL) complex’. A repair can be attempted for acute SLI presenting within 6 weeks of injury. Reconstruction is the mainstay of treatment for chronic SLI without degenerative changes. Multiple repair techniques have been described which include capsulodesis and tenodesis procedures. The clinical outcomes of the techniques have improved over the years. However, a common problem of all these techniques is the lack of long-term data on the outcomes and deteriorating radiological parameters over time. SLI staging is an important factor to be considered in choosing the reconstruction techniques for a better outcome. Currently, there is a trend towards more biological and less invasive techniques. Regardless of the technique, it is important to preserve the nerve supply of the dorsal capsuloligamentous structures of the wrist. Arthroscopic techniques being minimally invasive have the advantage of less collateral damage to the capsuloligamentous structures. Rehabilitation involves a team approach where a protected dart thrower’s motion is allowed after a period of immobilization. Strengthening SL-friendly muscles and inhibiting SL-unfriendly muscles is a key principle in rehabilitation.

## Introduction

The wrist is an intricate structure composed of eight carpal bones arranged in two rows with the scaphoid connecting the distal and proximal rows. The proximal row is an intercalated segment where no tendons attach. The distal carpal row bones are tightly bound to each other and function as a single unit. Ligaments are crucial for the stability of this uniquely complex structure. Hence, ligament injuries can lead to instability, abnormal mobility, joint loading, and degenerative changes.

Scapholunate instability (SLI) is the most common carpal instability pattern. While static SLI can be diagnosed by a combination of routine radiography, CT arthrography or MRI arthrography dynamic SLI can pose a diagnostic challenge. Untreated SLI can lead to degenerative changes of the wrist named ‘scapholunate advance collapse-SLAC’. While there are multiple repair and reconstruction techniques described in the literature, with improving clinical outcomes. In this article, we review the surgical anatomy, biomechanics, diagnosis, and treatment options for SLI without arthritic changes.

## Surgical Anatomy

The wrist is anatomically developed to maintain the precision and power of the human hand.

The distal carpal row is bound to each other with intercarpal ligaments and moves in response to the long wrist and finger tendons acting on the metacarpals. The only tendons attached to the distal carpal row are the slip of flexor carpi radialis (FCR) to the trapezium and the slip of flexor carpi ulnaris (FCU) to the hamate. The pisiform is a sesamoid bone within the FCU.

The proximal row consisting of the scaphoid, lunate, and triquetrum has no tendons inserted and is hence named the ‘intercalated segment’. The proximal row motion is directed by the moments transmitted from the distal row. The stability of this system depends largely on the ligamentous stabilisers with some support from the dynamic muscular stabilisers. A complex of dorsal, intrinsic and extrinsic ligaments with the dorsal capsule stabilises the proximal pole of the scaphoid to the lunate.

The scapholunate interosseous ligament (SLIL) is a C-shaped intrinsic ligament that attaches along the dorsal, proximal, and volar margins of the SL joint [1]. The dorsal component is 3–4 mm thick with transversely arranged collagen fibres. The volar component is approximately 1 mm thick, and the proximal component consists of fenestrated fibrocartilage. The scapho-trapezio-trapezial (STT) ligaments stabilise the distal pole of the scaphoid to the distal carpal row.

The important extrinsic carpal ligaments for scapholunate stability (SLI) are the dorsal inter-carpal ligament (DIC), dorsal radio-carpal ligament, long and short radio-lunate ligaments (LRL and SRL). Evidence from recent MRI studies shows that 58% of the patients with SLIL injury have concurrent DIC or DRC injury [2]. Therefore, pathoanatomically SLI is a multi-ligament injury.

**Fig. 1:**
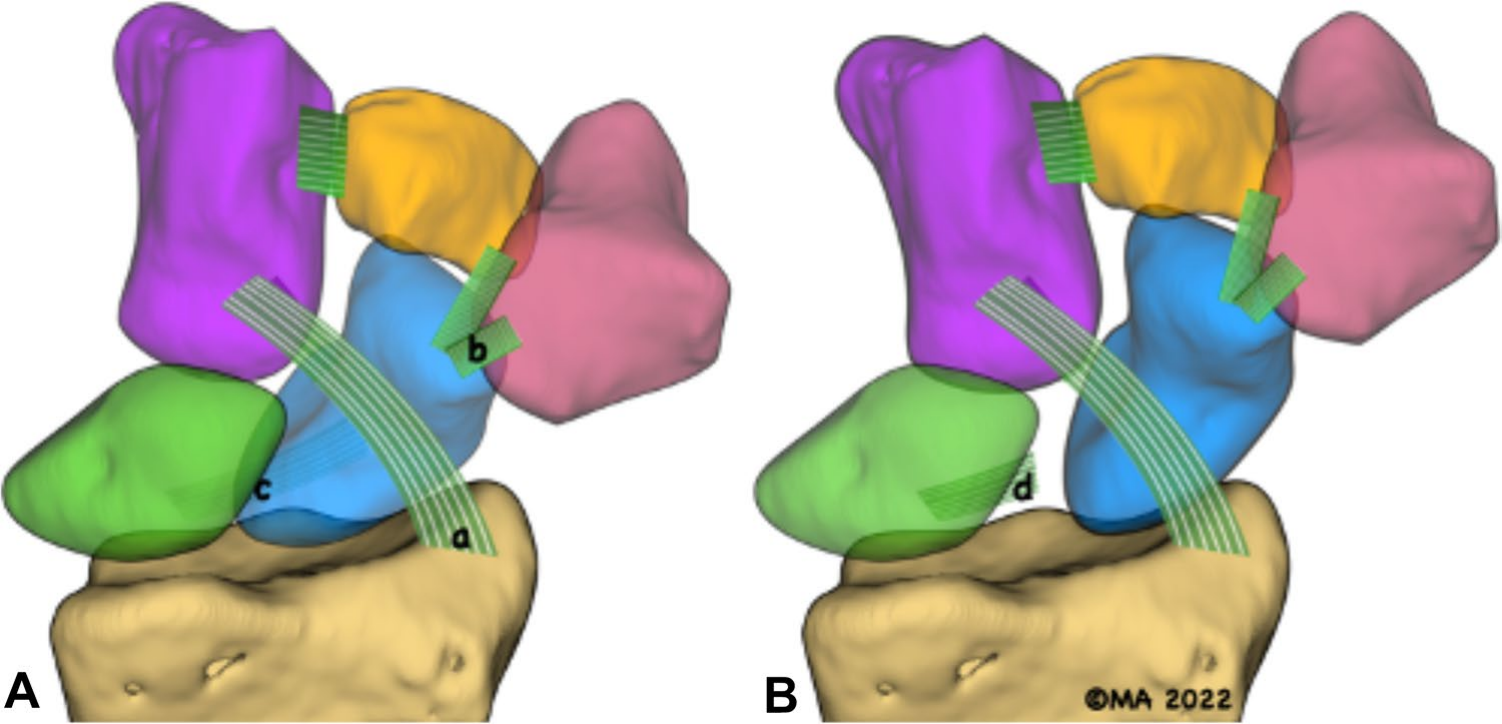
Ligaments that stabilise the scaphoid. **A**. Normal wrist **B**. Scapholunate instability wrist. *a*. Radioscaphocapitate (RSC) ligament. *b*. Scapho-trapezio-trapezoid (STT) ligaments. *c*. Diagrammatic representation of dorsal scapholunate ligament complex. *d*. Disrupted dorsal scapholunate ligament complex leading to dissociation of the scaphoid from the proximal carpal row. Image copyrights Melanie Amarasooriya

Dorsal capsule scapholunate septum (DCSS) is another anatomic structure that stabilises the scapho-lunate articulation [3]. Anatomy and the role of DCSS have been thoroughly studied and documented in the literature. The term “scapholunate ligament complex’ is widely used to describe the dorsal capsuloligamentous structures that stabilise the scapholunate interval [4]. The clinical syndrome and radiological pattern of SLI develop following the disruption of the ‘dorsal scapholunate complex’ (Fig. 1).

## Biomechanics

The wrist can maintain its stability through a wide range of motion and loading. The proximal row being an intercalated segment with no tendons attached, the stability was believed to be solely dependent on the articulations and the ligaments [5]. Historically the SLIL was believed to be the primary stabiliser of the SL joint [6]. However, there is mounting evidence that the dorsal intercarpal ligament (DIC) is equally or even more important in maintaining the scapholunate stability [7]. Cadaveric studies have proven that carpal instability defined by dorsal intercalated segmental instability (DISI) is unlikely to occur without injury to extrinsic carpal ligaments [8, 9].

While the ligamentous stability is undisputed, it is later reported that dynamic stability is maintained by the muscles and the tendons [10–12] and proprioception plays an important role in carpal stability [13]. When the injury cannot be compensated by the identified self-balancing mechanisms, carpal kinematics are disrupted, and the carpus becomes unstable.

Once the dorsal scapholunate ligament complex is compromised beyond the capacity of self-balancing mechanics of SL-friendly tendons, the scaphoid flexes, pronates and the lunate extends; scapholunate diastasis develops. The scaphoid behaves as a distal carpal row bone being subjected to unopposed forces from the radial distal carpal row. The proximal row dissociates at the scapholunate articulation, leading to abnormal kinematics and abnormal load transfer [14, 15]. The scapholunate unfriendly muscle extensor carpi ulnaris (ECU) can further accentuate the distal row pronation [16, 17]. A predictable pattern of arthritis develops following SLI [18, 19].

## Scapholunate Instability

Clinically, the SLI wrist is symptomatic during mechanical and load-bearing activities, demonstrating abnormal kinematics during wrist motion. The symptoms range from pain to sudden shifts or clunks during wrist motion. The instability may be static or dynamic depending on abnormal carpal positioning in a stress radiograph.

## Clinical Examination

An acute SL injury may present with pain, diffuse swelling, and tenderness localized over the dorsal wrist. There may be negative X-rays despite the wrist effusion. The identification of hemarthrosis implies an underlying ligament injury. Subacute injuries (1–6 weeks) present with painful clicking with wrist movement, decreased grip strength, and tenderness over the SL interval. Watson’s scaphoid shift test demonstrates the proximal scaphoid subluxation from the scaphoid fossa of the radius. While known for decades as a sign of scaphoid instability, the sensitivity and specificity of the scaphoid shift test are around 60–70% [20, 21]. The prevalence of positive scaphoid shift test in the uninjured population was reported to be as high as 32% [22].

## Classification

A clinically applicable classification of SLI was first developed by William Geissler [23]. This classification is based on wrist arthroscopy findings. Dreant and Dautel developed another classification in 2003 based on wrist arthroscopy to quantify the arthroscopic grading of SLI [24]. Currently, Garcia Elias’s classification which is a stepwise algorithm of management is widely used in clinical practice and research settings (Table 1). The management algorithm is based on defining pathoanatomy. Stages 1–2 indicate either the SLIL is intact or easily repairable. Stage 3 indicates scaphoid malalignment. Stage 04 considers the lunate uncovering index. Stage 05 occurs in the presence of the scaphoid and lunate malalignment which is easily reducible. Stage 06 is when the malalignment is chronic so that the reduction is difficult but still the cartilage is normal. It is likely that in stage 02 dorsal SLIL is injured. However, with advanced staging, one or more other critical stabilisers are likely to be injured, such as DIC, DRC, STT or the volar radio-lunate ligaments.

**Table 1:** Garcia-Elias classification of scapholunate instability (Garcia-Elias et al., 2006)

SLD stage	1	2	3	4	5	6
Is there a partial rupture with a normal dorsal SL ligament?	Yes	No	No	No	No	No
If ruptured, can the dorsal SL ligament be repaired?	Yes	Yes	No	No	No	No
Is the scaphoid normally aligned (radio-scaphoid angle < 45°)?	Yes	Yes	Yes	No	No	No
Is the carpal malalignment easily reducible?	Yes	Yes	Yes	Yes	No	No
Are the cartilages at both RC and MC joints normal?	Yes	Yes	Yes	Yes	Yes	No

European Wrist Arthroscopy Society (EWAS) has developed a further classification system for scapholunate ligament injury and correlated each stage to anatomo-pathological cadaveric findings (Table 2). They observe that the complete widening of the scapholunate gap occurs with a complete tear of SLIL and one or more extrinsic carpal ligaments.

**Table 2:** Arthroscopic EWAS (European Wrist Arthroscopy Society) Classification(Messina et al., 2013)

Arthroscopic stage (EWAS)	Arthroscopic testing of SLIOL from MC joint
I	No passage of the probe
II lesion of membranous SLIOL	Passage of the tip of the probe in the SL space without widening (stable)
III A partial lesion involving the volar SLIOL	Volar widening on dynamic testing from MC joint (Anterior laxity)
III B partial lesion involving the dorsal SLIOL	Dorsal SL widening on dynamic testing (Posterior laxity)
III C complete SLIOL tear, joint is reducible	Complete widening of SL space on dynamic testing, reducible with removal of probe
IV complete SLIOL with SL gap	SL gap with passage of the arthroscope from MC to RC joint No radiographic abnormalities
V	Wide SL gap with passage of the arthroscope through SL joint Frequent X-ray abnormalities such as an increased SL gap, DISI deformity

SLIOL: scapholunate interosseous ligament. MC: midcarpal. RC: radiocarpal. RSC: radio-scapho-capitate. LRL: long radiolunate. DIC: dorsal intercarpal ligament. SL: scapholunate. TH: triquetro-hamate. ST: scaphotrapezial. DRC: dorso radiocarpal. DISI: dorsal intercalated segmental instability

## Epidemiology

The exact incidence of SLI is unknown. Early literature quotes that 5% of sprained wrists are complicated by SLI [25]. This is usually following significant wrist trauma. However, there are multiple reports that patients with diagnosed SLI in one wrist have bilateral abnormal kinematics [26, 27]. Whether an injury occurs in people susceptible to SLI is yet to be resolved.

## Diagnosis

**Fig. 2:**
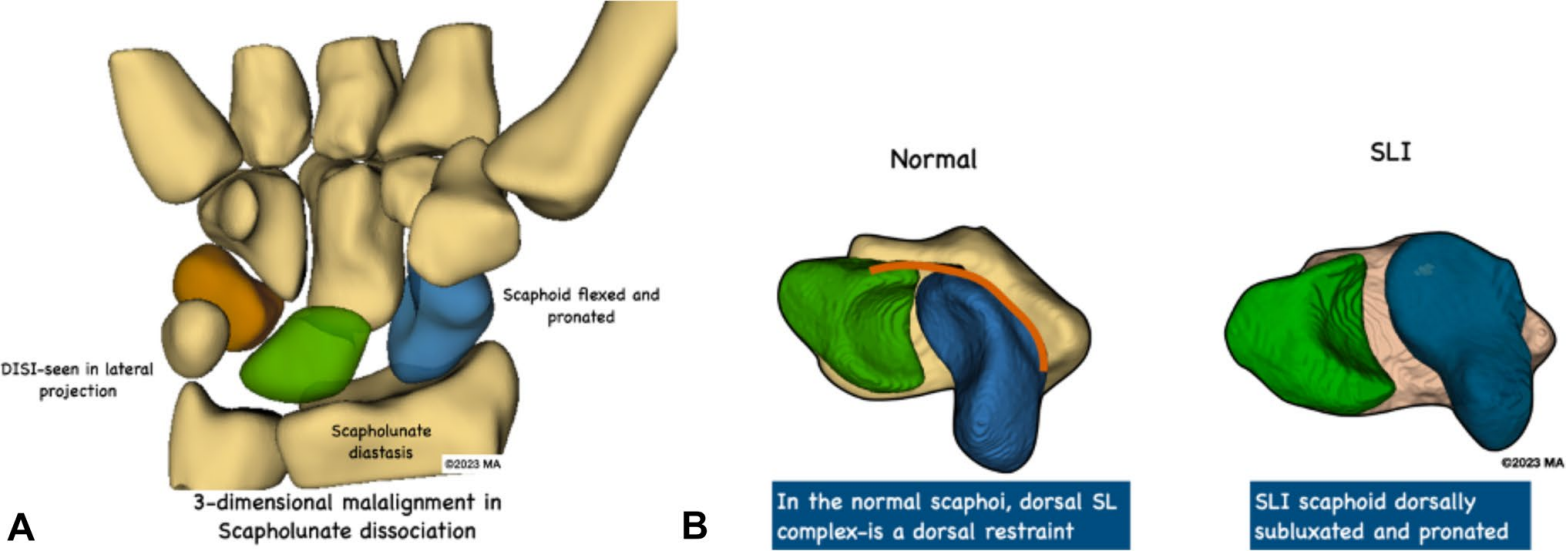
The three-dimensional malalignment in scapholunate instability. **a**. The SLI scaphoid is flexed and pronated. The scapholunate gap is wider. **b**. Compared to the normal scapholunate articulation in a distal view the SLI scaphoid is pronated and dorsally subluxated. The lunate is extended and the scapholunate gap is wide. Note that the normal and the SLI wrists in **b** are in radial deviation. Image copyrights Melanie Amarasooriya

**Fig. 3:**
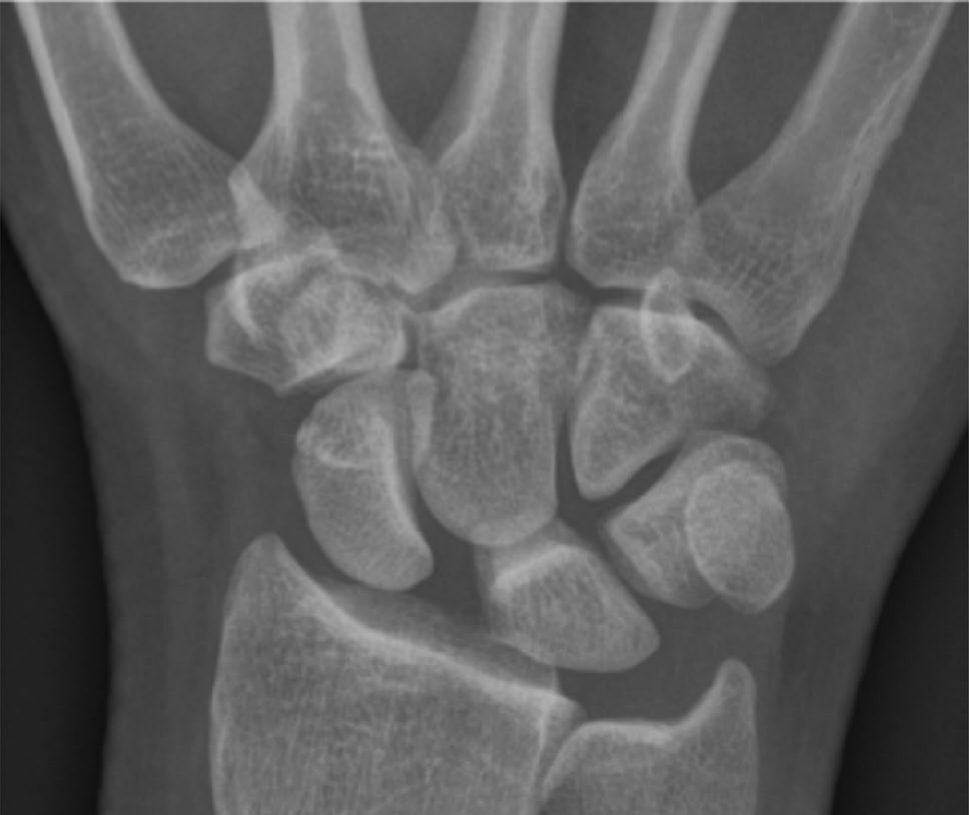
Posteroanterior view of the wrist. The scaphoid is flexed demonstrating the signet ring appearance, there is a wide scapholunate interval

Despite being the most common carpal instability SLI is often missed [28]. It is important to understand the 3-D malalignment of SLI for better diagnosis (Fig. 2). A summary of diagnostic modalities, potential findings, sensitivities and specificities in diagnosing SLI is given in Table 3. The current preliminary diagnostic workup includes the standard postero-anterior and lateral X-rays [29] and contralateral wrist radiographs for comparison. The scaphoid flexion, lunate extension, scapholunate diastasis and dorsal scaphoid translation are the common findings that are identifiable using static X-rays [5](Fig. 3). A scapholunate angle over 70° and lunate extension over 15° are pathological and suggestive of SLI.

**Table 3:** Investigations used in diagnosing scapholunate instability

Investigation	Findings	Sensitivity/specificity
Plain X-ray (posteroanterior and lateral)	Scaphoid flexion Dorsal intercalated segmental instability (DISI) Scapholunate diastasis Increased scapholunate angle (> 70°) Dorsal subluxation of the proximal pole	Sensitivity 81% Specificity 80% (Sulkers et al., 2014)
Stress X rays (clenched fist, pencil grip, twist x ray)	Worsening of SL diastasis with stress	NA
Dynamic fluoroscopy	Worsening of SL diastasis with stress and movement (SL gap increases in ulnar deviation) Dorsal subluxation of proximal pole with radial deviation	Sensitivity 90% Specificity 97% (Sulkers et al., 2014)
CTA-CT arthrogram	Direct visualisation the SL ligament/ partial or full thickness tears Contrast leakage between compartments suggestive of SL injury radiocarpal opacification-for cartilage assessment	Sensitivity 85–97% Specificity 79–97% (Bille et al., 2007)
Dynamic CT	Worsening SL gap (with movement and stress) Dynamic dorsal subluxation of the scaphoid	NA
Magnetic resonance imaging (MRI)	Partial or complete tears in SLIL Tears in DIC and DRC and other carpal ligament injuries Cartilage assessment	Sensitivity 75.7% Specificity 97.1% (Hafezi-Nejad et al., 2016)
Magnetic resonance arthrography (MRA)	In addition to features seen in MRI better appreciation of ligament tears granulation tissue associated with fibrous scar tissue infiltration of the injured ligament Contrast extravasation from radiocarpal or midcarpal compartment	Sensitivity 82% and specificity 93% (Hafezi-Nejad et al., 2016)

Various stress views which are modifications of the clenched fist view are helpful in the diagnosis of dynamic SLI. The twist X-ray, clenched fist, and pencil grip view are some of the common alternatives [30]. Dynamic fluoroscopy enables the wrist to be moved through a range of motion and identify dynamic changes in real-time. Dynamic fluoroscopy has proven to have a sensitivity of 90%, a specificity of 97% and a diagnostic accuracy of 93% [31] in diagnosing SLI.

Static computed tomography (CT) scans are commonly used to diagnose wrist pathology. When combined with arthrography as in CT arthrogram can be very accurate. The diagnostic accuracy of CTA is found to be better than conventional MRI in detecting SLIL tears in a cadaveric model when using oblique and axial planar sections [32]. CTA is recognized to have a sensitivity of 94% and specificity of 86% in detecting SLIL tears when arthroscopy is considered the gold standard diagnostic test [33]. The chondral loss also can be assessed by CTA. A meta-analysis comparing 3T MRI, MRA and 1.5T MRI for diagnostic accuracy of SLI found a sensitivity of 82% and specificity of 93% for MRA compared to arthroscopy or gross pathology as standard of reference. MRA had the best diagnostic accuracy and 3T MRI had the best specificity in detecting SLIL injuries [34].

While it is a fact that SLI is complicated by extrinsic carpal ligament injuries, validated scientific evidence on differentiation of partial incomplete or complete tears is limited. With the available limited evidence, MRA is reported to provide the most accurate information on the integrity of the DCSS. Of all the diagnostic parameters described only variable linked to patient outcome is the dorsal scaphoid translation (DST) [35].

Dynamic MRI and dynamic CT have created a lot of research interest in recent years; however, the place of these investigations in the diagnosis of SLI is not established due to limited availability and different study protocols used in the different centres [29].

## Reconstruction Options

There is a myriad of reconstruction options for SLI, but not an established gold standard. The outcomes are varied with almost all the techniques having proven medium-term clinical results [36, 37]. Long-term clinical results are limited and the worsening radiographic parameters over the medium term is not uncommon.

## Acute Repair

There is evidence that repair of acutely presenting SLI leads to a good outcome, as the healing potential of the ligament in the acute stage is satisfactory [38–41]. The SL ligament is mostly avulsed from the scaphoid, or the lunate with occasional mid-substance tears [42]. The DIC, however, is commonly found to be avulsed from the lunate [43].

Open surgery for acute repair can be performed through Berger’s ligament-preserving dorsal capsulotomy to access the dorsal SLIL [44]. However, for an acute repair, the author’s preference is the window approach which gives adequate exposure with less soft tissue compromise [45]. A mattress suture is placed on the avulsed SLIL/DIC complex and brought down to the footprint on the scaphoid or the lunate using suture anchors. The author’s experience is that tensionable anchors can be useful in getting the ligament to be seated on the anatomical footprint. Minami et al. reported that after 03 years from acute repair that despite the good clinical results the scaphoid malrotation advanced radiologically. Combining acute repair with dorsal capsulodesis has proven to improve the results [46, 47].

Once the acute stage has passed the chances of the ligament healing are unpredictable. It is common practice to attempt reconstruction in late presentations for SLI presenting after 6 weeks from injury. However, the timeline for this decision is arbitrary and based on individual surgeons’ preferences.

## Reconstruction

Repair or reconstruction techniques are broadly categorised into two, capsulodesis and tenodesis procedures.

## Capsulodesis

Capsulodesis alone has a variable clinical and radiological outcome. Blatt initially described attaching a 1 cm wide dorsal capsular flap containing the DRC ligament rotated based on the distal radius to the distal scaphoid [48]. Slater modification (Mayo technique) of advancing a flap of DIC onto the distal scaphoid is biomechanically stronger than the original Blatt’s technique [49]. Results of capsulodesis procedures report no major complications; however, there is a worsening of radiological parameters over the years. Micicoi et al. reported an average 54-month follow-up for a cohort of 120 patients following Mayo technique capsulodesis. They found improvement in all three clinical, functional and radiological outcome measures. However, it is worth noting that 96 patients of their cohort belonged to pre-dynamic and dynamic SLI. Other studies on the same technique reported deterioration of the radiological parameters over time [50–52]. Thus, modified capsulodesis procedures have a place in early-stage SLI (pre-dynamic and dynamic).

## Bone–Ligament–Bone Graft

Bone–ligament–bone graft as proposed by Weiss is another option for early grades of SLI [53]. This initial description used a graft from the dorsal distal radius with variable results. Other graft options were described in later literature. Morrell and Weiss reported on long-term follow-up bone–retinaculum–bone autografts over an average of 11.9 years and concluded that there was a moderate deterioration of radiological parameters and fields [54, 55]. The authors recommend this technique be used only in cases with a reducible SL gap, and anatomic reduction is needed before positing the graft.

## Tenodesis

There are multiple non-anatomical tenodesis techniques based on extensor carpi radialis brevis (ECRB) and flexor carpi radialis (FCR). Georgiou Brunelli introduced the Brunelli technique where a strip of FCR based on the volar index metacarpal is drawn across the scaphoid from volar to dorsal and anchored to the dorsal radius [56]. Conceptually Brunelli believed that the pathoanatomy of SLI was STT ligament insufficiency. His methods aim to stabilise the scaphoid on the radius. However, the most common complication was wrist stiffness as two joints (STT and the radiocarpal) were tenodesed with one strip of FCR. Van den Abbeele et al. modified this technique so that the distal end is woven around the DRC ligament and attached on to the lunate [57]. Garcia-Elias further modified this technique by making the scaphoid tunnel more oblique and attaching the tendon to the dorsal lunate with a suture anchor in the 3-ligament tenodesis technique [58].

There are multiple clinical studies proving good mid-term clinical outcomes following the 3-LT technique [58]. However, recurrence of the abnormal radiological findings in the medium term is not uncommon [59]. A further modification using tensionable anchors while recreating the STT, dorsal SLIL, DIC and DRC has been reported to yield satisfactory clinical outcomes [60].

## Reduction and Association of Scapholunate Interval and Scapholunate Axis Method

Reduction and Association of the scapholunate interval (RASL) procedure [61] uses a headless compression screw to create a fibrous union between the scaphoid and the lunate. Once the fibrous union occurs, the screw is meant to be removed. The scapholunate axis method (SLAM) is also based on the same principle but not needing hardware removal [62]. While it is intuitive to think that a central axis for the scapholunate articulation will restore the kinematics, the biomechanical evidence is to the contrary. The scapholunate rotation axis during various wrist positions is very variable [63]. Restoring a rigid SL axis hence can lead to hardware failure, loosening and tunnel enlargement, which has been reported in clinical results [64].

## All Dorsal Reconstructions

In recent years, the focus is shifted to all dorsal SL reconstructions. There is a number of all dorsal reconstructions that include SLICL, internal brace technique and arthroscopic dorsal reconstructions.

Arthrex® internal brace® is an all-dorsal reconstruction technique which is graft augmented [65]. While there is biomechanical evidence that it adds to the SLIL repair strength, whether it recreates natural carpal kinematics is doubtful [66].

Athlani et al. has proposed an all-dorsal reconstruction, Scapholunate and Intercarpal Ligamentoplasty (SLICL). This technique aims to recreate the dorsal component of the SLIL and DIC ligament. A free palmaris graft is anchored to a bone tunnel in the dorsal scaphoid with an interference (IF) screw, then to a trench in the lunate with a suture anchor, to a bone tunnel in the triquetrum with an IF screw and back to a bone tunnel in the scaphoid with IF screw. While there are multiple bone tunnels, the authors mention that as they were not trans-osseous, tunnel-related complications could be less. The authors report good clinical and radiological correction (scapholunate gap, scapholunate angle) compared to the 3-LT technique following 26 procedures with an average 36-month follow-up. They have also noted 04 rapid recurrences of SLI following k-wire removal postoperatively. This was attributed to poor case selection, as these patients were stage 05 SLI in the Garcia–Elias classification. The authors recommend the technique for ‘easily reducible’ SLI. This further strengthens the fact that patient selection for each technique is an important factor to get a good outcome.

Based on the theory of the importance of DIC the RADICAL procedure (Repair/Augmentation of Dorsal Intercarpal Ligament) was introduced by Williams et al. [67]. This involves extrinsic ligament re-insertion with arthroscopic or mini-open techniques into the dorsal lunate. This procedure is more biological, less invasive and preserves native capsuloligamentous structures. Authors recommend this technique for DIC/DRC injuries associated with partial SLIL and long-term results are awaited.

## Volar and Dorsal Reconstructions

The scapholunate interosseous ligament has a volar component which is believed to be important in rotational stability. Henry et al. recommended recreating the volar component of the ligament with an FCR tendon [68]. While the conceptually appealing author has only described a single case with an 08-year follow-up. ANAFAB-Anatomic front and back repair aim to restore the scapho-lunate interosseous ligament and potential volar carpal ligaments that may have been injured [69]. The technique is based on a distally based FCR strip augmented with synthetic tape, drawn across a bone tunnel in scaphoid from volar to dorsal and then dorsal to volar across a bone tunnel in the lunate. The final step is to anchor the graft to the volar radius recreating the volar carpal ligaments. A minimum 2-year follow-up of 10 patients reported good clinical functional and radiological results [69].

## Biomechanics Analysis of Reconstruction Techniques

A recent study by Burnier et al. proposed that ANAFAB better recreates the scapholunate angle and radio-lunate angle and was the only technique to significantly reduce the dorsal scaphoid translation. In Burner’s biomechanical study, the 3 LT does reduce the SLG and SLA [70] and the RASL reduces the scapholunate gap compared to the 3LT. The RASL reduces the scapholunate gap compared to the 3LT and ANAFAB. The conclusion drawn comparing three techniques, RASL, 3-LT and ANAFAB was that each technique has its own strengths and specific place in SLI reconstruction. ANAFAB reconstructed most of the variables assessed.

Chae et al. compared the biomechanical strength of 360° SLIL reconstruction only using an artificial material (AM), double dorsal limb (DDL) SLIL reconstruction only using AM, and the modified Brunelli technique (MBT) with the ligament. The authors reported that all three reconstruction techniques could restore the dorsal SL distance; however, only the 360° SLIL technique restored the volar SL distance in the wrist extension [71]. While this is biomechanically an important finding, experience in ACL reconstruction in the knee suggests that the use of artificial material has to be further evolved to be more biocompatible and of similar strength to the native ligament [72].

## Role of Arthroscopy

Arthroscopy is undoubtedly the gold standard in diagnosing and classifying scapholunate instability. While directly providing a visual appreciation of the injured ligaments, arthroscopy can also be used to demonstrate instability by eliciting scaphoid shift test [73]. Its role in repair and reconstruction continues to evolve.

Christopher Mathoulin has pioneered an all-dorsal arthroscopic reconstruction, which is minimally invasive with the least compromise to the soft tissue [74, 75]. His construct is based on the DCSS which is identified as a distinct structure important for SL stability. This ligament has the dorsal attachment between the DIC, the scaphoid-triquetral ligament, and the dorsal SL ligament. The SL ligament remnants are also sutured to each other and the dorsal capsule. Arthroscopic repair is only recommended when a ligament remnant exists on both the lunate and scaphoid. The authors report the outcomes of 221 procedures with a mean follow-up of 40 months. The mean grip strength was 93% of the contralateral side. They observed an increase in flexion by 11° and extension by 14° compared to the preoperative range of motion. The post-operative DASH score was 09 compared to a preoperative value of 47. DISI, however, was uncorrected in 19% of the cases. The long-term results of this procedure are yet to be published.

Arthroscopic ligament reconstruction by tenodesis has been attempted with success by some authors. Fernando Corella’s all-arthroscopic reconstruction of volar and dorsal SLI addresses SLI without damaging the dorsal wrist soft tissue envelope [76]. However, the authors comment that despite a significant improvement in the SL angle and the gap, the gap remained open in many patients with static instability.

The main advantage of arthroscopic repair is that it respects the innervation and the dorsal capsule. It is shown that the innervation of the wrist joint is important in adequate neuromuscular control [13, 77]. Therefore, it is prudent that nerve-sparing approaches are used either arthroscopically or when using open surgery so that the self-balancing mechanisms of the wrist are not jeopardized.

Over the last few decades, reconstruction techniques have evolved to result in better outcomes. Recently there is a trend towards more biological, less invasive reconstructions. Learning from knee ligamentization, it is suggested that ligament sparing demonstrated a trend toward improvements in vascularity, mechanoreceptors, and biomechanics that lessens in significance over time [78].

## Natural History

Kinetics, kinematics and the self-stabilising role of tendons continue to be studied. Watson has suggested that untreated scapholunate instability progresses into a predictable pattern of arthritis ending in the SLAC wrist [79]. However, the natural history of dynamic SLI is less convincing [80].

## Rehabilitation

The overall goal of scapholunate repair or reconstruction is to recreate normal kinetics and kinematics of the carpus preventing degenerative changes. The overall goal of rehabilitation of SL injury is to protect the injured or healing structures while promoting mobility for better functional outcomes. Immobilization would help protection of healing tissue but when prolonged has the risk of causing stiffness. Therefore, an ideal rehabilitation regimen should involve protected motion and strengthening exercises that do not affect the healing structures.

Wrist motion from radial extension to ulnar flexion, along the dart throwers plane, is known to have the least motion in the proximal carpal row [81]. When the wrist is moving in dart throwers motion, the midcarpal joint moves around a stable proximal carpal row. Therefore, a gentle protected range of motion in the dart thrower’s plane is believed to be safe in the rehabilitation of post-SLI reconstruction patients [12]. Gentle-protected DTM has successfully been used in clinical series [82]. Based on this concept various commercial hinged wrist orthoses are available [83, 84].

However, a 4D CT study by Garcia Elias et al. states that in SLI wrists the scapholunate gap widens with DTM [85]. In addition, the exact coupling ratio of flexion–extension to radioulnar deviation for various tasks varies [86]. The coupling ratio of flexion–extension to radioulnar deviation for a ‘motionless’ proximal row for individual patients may be variable. Considering these facts, only a protected and limited range of motion is advised in the early postoperative period. Splints in the early postoperative stage stabilize the SL joint by limiting its motion. Many splints and custom-designed orthoses are available to limit wrist motion to a DTM plane and minimize the stress of SLIL, lunate and scaphoid movements. Wrist circumduction for rehabilitation is performed by an elliptical movement that combines the position of the wrist in all directions. The circumduction envelope is the three-dimensional space that is generated by this motion. The rehabilitation maximizes the wrist circumduction and couples motion path required for activities and exercises that challenge the extremes of wrist motion (wrist maze exerciser, wrist wand, and baton).

Neuromuscular control of the wrist is important. The dorsal carpal ligaments are richly innervated with proprioceptive nerve endings [13, 87]. On an unconscious level, the mechanoreceptors embedded in the ligaments detect joint position change. These signals are carried via afferent pathways to the spinal cord to elicit reflexes to increase efferent muscular input to augment joint stability. On a conscious level, anticipatory planning and execution of voluntary joint control may involve the central nervous system [88].

The proprioceptive nerve endings on carpal ligaments recruit the self-protective neuromuscular mechanisms. Isolated and isometric contraction of ECRL and APL causes a supinating moment on the scaphoid, reducing the scaphoid onto the anatomical alignment [17, 89]. These are the muscles that drive the radial extension of the DTM. Isolated isometric contraction of the FCR will also supinate the scaphoid with pronation of the triquetrum. This knowledge is used to create rehabilitation programs for scapholunate instability. Therefore, the recommended muscle strengthening exercise for SL dissociation is an isometric co-contraction of the ECRL/APL muscles and flexor carpi radialis because these muscles act to supinate the scaphoid, thus minimizing the gap. On the other hand, ECU and FCU can worsen the SL gap by pronating the scaphoid. Hence, a rehabilitation program should avoid recruiting the ECU and FCU.

## Conclusion

Despite being the most common carpal instability SLI can be challenging in terms of diagnosis and treatment. The pathoanatomy of SLI involves extrinsic carpal ligaments in addition to SLIL. It is important to identify SLIL as a 3-dimensional malalignment and instability of the carpus due to a multi-ligament injury that includes SLIL and extrinsic ligaments. Maintaining a high degree of suspicion in clinical cases and the use of appropriate imaging modalities can improve diagnostics. Arthroscopy remains the gold standard for diagnosis.

Among many reconstruction techniques that have been proposed, there is no clear gold standard. All authors recommend using each technique for specific indications, respecting the stage of SLI. Patient selection for each technique is a key factor for a good outcome. Respecting soft tissue integrity and minimum collateral damage is helpful for healing. The repair or reconstruction should consider the SLI stage and respect the wrist's proprioceptive nerve supply. When we extrapolate the principles of fracture fixation to ligament reconstruction, anatomic reduction of midcarpal and radiocarpal joints is an essential step followed by stable fixation. Rehabilitation should aim for an early protected range of motion until the repair heals.

## Limitations

This is a review of the SL instability without arthritic changes. The primary goal is to reduce pain, restore function and delay the onset of the degenerative changes by restoring the carpal alignment and improving load distribution. The treatment for SL instability with arthritic changes is different and beyond the scope of this review.

## References

[1] Berger RA, Blair WF, Crowninshield RD, Flatt AE (1982). The scapholunate ligament. Journal of Hand Surgery (American Volume).

[2] Özkan S, Kheterpal A, Palmer WE, Chen NC (2019). Dorsal extrinsic ligament injury and static scapholunate diastasis on magnetic resonance imaging scans. Journal of Hand Surgery (American Volume).

[3] Tommasini Carrara de Sambuy M, Burgess TM, Cambon-Binder A, Mathoulin CL (2017). The anatomy of the dorsal capsulo-scapholunate septum: a cadaveric study. Journal of Wrist Surgery.

[4] Slutsky DJ (2013). The scapholunate ligament complex (SLLC). Journal of Wrist Surgery.

[5] Linscheid RL, Dobyns JH, Beabout JW, Bryan RS (1972). Traumatic instability of the wrist Diagnosis, classification, and pathomechanics. Journal of Bone and Joint Surgery..

[6] Short WH, Werner FW, Green JK, Masaoka S (2002). Biomechanical evaluation of ligamentous stabilizers of the scaphoid and lunate. Journal of Hand Surgery (American Volume).

[7] Raja S, Williams D, Wolfe S, Couzens G, Ross M. New Concepts in Carpal Instability. In: *Wrist and Elbow Arthroscopy with Selected Open Procedures.* Springer, Cham. 2022:173–185.

[8] Pérez AJ, Jethanandani RG, Vutescu ES, Meyers KN, Lee SK, Wolfe SW (2019). Role of ligament stabilizers of the proximal carpal row in preventing dorsal intercalated segment instability: a cadaveric study. Journal of Bone and Joint Surgery American Volume.

[9] Elsaidi GA, Ruch DS, Kuzma GR, Smith BP (2004). Dorsal wrist ligament insertions stabilize the scapholunate interval: Cadaver study. Clinical Orthopaedics and Related Research.

[10] Linscheid RL, Dobyns JH (2002). Dynamic carpal stability. Keio Journal of Medicine.

[11] Salvà-Coll G, Garcia-Elias M, Llusá-Pérez M, Rodríguez-Baeza A (2011). The role of the flexor carpi radialis muscle in scapholunate instability. Journal of Hand Surgery (American Volume).

[12] Salva-Coll G, Garcia-Elias M, Leon-Lopez MT, Llusa-Perez M, Rodríguez-Baeza A (2011). Effects of forearm muscles on carpal stability. Journal of Hand Surgery (European Volume).

[13] Hagert E, Persson JK, Werner M, Ljung BO (2009). Evidence of wrist proprioceptive reflexes elicited after stimulation of the scapholunate interosseous ligament. Journal of Hand Surgery (American Volume).

[14] Omori S, Moritomo H, Omokawa S, Murase T, Sugamoto K, Yoshikawa H (2013). In vivo 3-dimensional analysis of dorsal intercalated segment instability deformity secondary to scapholunate dissociation: A preliminary report. Journal of Hand Surgery (American Volume).

[15] Amarasooriya M, MacLean S, Bain GI, Bhatia DN, Bain GI, Poehling GG, Graves BR (2022). Clinical biomechanics of the wrist. Arthroscopy and endoscopy of the elbow, wrist and hand: surgical anatomy and techniques.

[16] Salva-Coll G, Garcia-Elias M, Hagert E (2013). Scapholunate instability: Proprioception and neuromuscular control. Journal of Wrist Surgery.

[17] Leon-Lopez MM, Garcia-Elias M, Salva-Coll G, Llusa-Perez M, Lluch-Bergada A (2014). Muscular control of scapholunate instability. An experimental study. Revista Española de Cirugía Ortopédica y Traumatología.

[18] Watson HK, Ryu J (1986). Evolution of arthritis of the wrist. Clinical Orthopaedics and Related Research.

[19] Watson HK, Weinzweig J, Zeppieri J (1997). The natural progression of scaphoid instability. Hand Clinics.

[20] Schmauss D, Pöhlmann S, Weinzierl A (2022). Relevance of the scaphoid shift test for the investigation of scapholunate ligament injuries. Journal of Clinical Medicine.

[21] LaStayo P, Howell J (1995). Clinical provocative tests used in evaluating wrist pain: A descriptive study. Journal of Hand Therapy..

[22] Easterling KJ, Wolfe SW (1994). Scaphoid shift in the uninjured wrist. Journal of Hand Surgery (American Volume)..

[23] Geissler WB (2006). Arthroscopic management of scapholunate instability. Chirurgie de la Main.

[24] Dreant N, Dautel G (2003). Development of a arthroscopic severity score for scapholunate instability. Chirurgie de la Main.

[25] Jones WA (1988). Beware the sprained wrist The incidence and diagnosis of scapholunate instability. Journal of Bone and Joint Surgery: British Volume.

[26] Picha BM, Konstantakos EK, Gordon DA (2012). Incidence of bilateral scapholunate dissociation in symptomatic and asymptomatic wrists. Journal of Hand Surgery (American Volume)..

[27] Crisco JJ, Pike S, Hulsizer-Galvin DL, Akelman E, Weiss AP, Wolfe SW (2003). Carpal bone postures and motions are abnormal in both wrists of patients with unilateral scapholunate interosseous ligament tears. Journal of Hand Surgery (American Volume)..

[28] Pietsch E (2019). Injuries to the Scapholunate Ligament: How Often do we Miss it?. EC Orthopaedics..

[29] Dietrich TJ, Toms AP, Cerezal L (2021). Interdisciplinary consensus statements on imaging of scapholunate joint instability. European Radiology..

[30] Sikora SK, Tham SK, Harvey JN (2019). The twist X-ray: A novel test for dynamic scapholunate instability. Journal of Wrist Surgery.

[31] Sulkers GSI, Schep NWL, Maas M, van der Horst CMAM, Goslings JC, Strackee SD (2013). The diagnostic accuracy of wrist cineradiography in diagnosing scapholunate dissociation. Journal of Hand Surgery (European Volume)..

[32] Lee RK, Griffith JF, Ng AW (2017). Intrinsic carpal ligaments on MR and multidetector CT arthrography: Comparison of axial and axial oblique planes. European Radiology.

[33] Bille B, Harley B, Cohen H (2007). A comparison of CT arthrography of the wrist to findings during wrist arthroscopy. Journal of Hand Surgery (American Volume)..

[34] Hafezi-Nejad N, Carrino JA, Eng J (2016). Scapholunate interosseous ligament tears: diagnostic performance of 1.5 T, 3 T MRI, and MR arthrography—A systematic review and meta-analysis. Academic Radiology..

[35] Gondim Teixeira PA, De Verbizier J, Aptel S (2016). posterior radioscaphoid angle as a predictor of wrist degenerative joint disease in patients with scapholunate ligament tears. AJR. American Journal of Roentgenology.

[36] Chennagiri RJ, Lindau TR (2013). Assessment of scapholunate instability and review of evidence for management in the absence of arthritis. Journal of Hand Surgery (European Volume).

[37] Naqui Z, Khor WS, Mishra A, Lees V, Muir L (2018). The management of chronic non-arthritic scapholunate dissociation: A systematic review. Journal of Hand Surgery (European Volume).

[38] Beredjiklian PK, Dugas J, Gerwin M (1998). Primary repair of the scapholunate ligament. Techniques in Hand & Upper Extremity Surgery.

[39] Bickert B, Sauerbier M, Germann G (2000). Scapholunate ligament repair using the Mitek bone anchor. Journal of Hand Surgery (British volume).

[40] Rosati M, Parchi P, Cacianti M, Poggetti A, Lisanti M (2010). Treatment of acute scapholunate ligament injuries with bone anchor. Musculoskeletal Surgery.

[41] Minami A, Kaneda K (1993). Repair and/or reconstruction of scapholunate interosseous ligament in lunate and perilunate dislocations. Journal of Hand Surgery (American Volume)..

[42] Andersson JK, Garcia-Elias M (2013). Dorsal scapholunate ligament injury: A classification of clinical forms. Journal of Hand Surgery (European Volume).

[43] Raja S, Williams D, Wolfe SW, Couzens G, Ross M, Geissler WB (2022). New concepts in carpal instability. Wrist and elbow arthroscopy with selected open procedures: A practical surgical guide to techniques.

[44] Berger RA, Bishop AT, Bettinger PC (1995). New dorsal capsulotomy for the surgical exposure of the wrist. Annals of Plastic Surgery.

[45] Loisel F, Wessel LE, Morse KW, Victoria C, Meyers KN, Wolfe SW (2021). Is the dorsal fiber-splitting approach to the wrist safe? a kinematic analysis and introduction of the "window" approach. Journal of Hand Surgery (American Volume)..

[46] Minami A, Kato H, Iwasaki N (2003). Treatment of scapholunate dissociation: Ligamentous repair associated with modified dorsal capsulodesis. Hand Surgery.

[47] Lavernia CJ, Cohen MS, Taleisnik J (1992). Treatment of scapholunate dissociation by ligamentous repair and capsulodesis. Journal of Hand Surgery (American Volume)..

[48] Blatt G (1987). Capsulodesis in reconstructive hand surgery: dorsal capsulodesis for the unstable scaphoid and volar capsulodesis following excision of the distal ulna. Hand Clinics..

[49] Slater RR, Szabo RM, Bay BK, Laubach J (1999). Dorsal intercarpal ligament capsulodesis for scapholunate dissociation: Biomechanical analysis in a cadaver model. Journal of Hand Surgery (American Volume)..

[50] Moran SL, Ford KS, Wulf CA, Cooney WP (2006). Outcomes of dorsal capsulodesis and tenodesis for treatment of scapholunate instability. Journal of Hand Surgery (American Volume)..

[51] Gajendran VK, Peterson B, Slater RR, Szabo RM (2007). Long-term outcomes of dorsal intercarpal ligament capsulodesis for chronic scapholunate dissociation. Journal of Hand Surgery (American Volume)..

[52] Megerle K, Bertel D, Germann G, Lehnhardt M, Hellmich S (2012). Long-term results of dorsal intercarpal ligament capsulodesis for the treatment of chronic scapholunate instability. Journal of Bone and Joint Surgery. British Volume.

[53] Weiss AP (1998). Scapholunate ligament reconstruction using a bone-retinaculum-bone autograft. Journal of Hand Surgery (American Volume)..

[54] Soong M, Merrell GA, Ortmann FT, Weiss AP (2013). Long-term results of bone-retinaculum-bone autograft for scapholunate instability. Journal of Hand Surgery (American Volume)..

[55] Shin SS, Moore DC, McGovern RD, Weiss AP (1998). Scapholunate ligament reconstruction using a bone-retinaculum-bone autograft: A biomechanic and histologic study. Journal of Hand Surgery (American Volume)..

[56] Brunelli GA, Brunelli GR (1995). A new surgical technique for carpal instability with scapho-lunar dislocation. (Eleven cases). Annales de chirurgie de la main et du membre supérieur.

[57] Van Den Abbeele KLS, Loh YC, Stanley JK, Trail IA (1998). Early results of a modified Brunelli procedure for scapholunate instability. Journal of Hand Surgery..

[58] Garcia-Elias M, Lluch AL, Stanley JK (2006). Three-ligament tenodesis for the treatment of scapholunate dissociation: Indications and surgical technique. Journal of Hand Surgery (American Volume)..

[59] Athlani L, Pauchard N, Detammaecker R (2018). Treatment of chronic scapholunate dissociation with tenodesis: A systematic review. Hand Surgery and Rehabilitation..

[60] Bain GI, Watts AC, McLean J, Lee YC, Eng K (2015). Cable-augmented, quad ligament tenodesis scapholunate reconstruction. Journal of Wrist Surgery.

[61] Rosenwasser MP, Miyasajsa KC, Strauch RJ (1997). The RASL procedure: Reduction and association of the scaphoid and lunate using the Herbert screw. Techniques in Hand & Upper Extremity Surgery..

[62] Yao J, Zlotolow DA, Lee SK (2016). ScaphoLunate axis method. Journal of Wrist Surgery.

[63] Best GM, Mack ZE, Pichora DR, Crisco JJ, Kamal RN, Rainbow MJ (2019). Differences in the rotation axes of the scapholunate joint during flexion-extension and radial-ulnar deviation motions. Journal of Hand Surgery (American Volume)..

[64] Aibinder WR, Izadpanah A, Elhassan BT (2019). Reduction and association of the scaphoid and lunate: a functional and radiographical outcome study. Journal of Wrist Surgery.

[65] Kakar S, Greene RM (2018). Scapholunate ligament internal brace 360-degree tenodesis (SLITT) procedure. Journal of Wrist Surgery.

[66] Park IJ, Maniglio M, Shin SS, Lim D, McGarry MH, Lee TQ (2020). Internal bracing augmentation for scapholunate interosseous ligament repair: a cadaveric biomechanical study. Journal of Hand Surgery (American Volume)..

[67] Williams D, Raja S, Ross M, Couzens G, Wolfe SW, Geissler WB (2022). The RADICL procedure: repair/augmentation of dorsal intercarpal ligament. Wrist and elbow arthroscopy with selected open procedures: A practical surgical guide to techniques.

[68] Henry M (2013). Reconstruction of both volar and dorsal limbs of the scapholunate interosseous ligament. Journal of Hand Surgery (American Volume)..

[69] Sandow M, Fisher T (2020). Anatomical anterior and posterior reconstruction for scapholunate dissociation: Preliminary outcome in ten patients. Journal of Hand Surgery (European Volume).

[70] Burnier M, Jethanandani R, Pérez A, Meyers K, Lee S, Wolfe SW (2021). Comparative analysis of 3 techniques of scapholunate reconstruction for dorsal intercalated segment instability. Journal of Hand Surgery (American Volume).

[71] Chae S, Nam J, Park I-J, Shin SS, McGarry MH, Lee TQ (2022). Biomechanical analysis of three different reconstruction techniques for scapholunate instability: A cadaveric study. Clinics in Orthopedic Surgery..

[72] Legnani C, Ventura A, Terzaghi C, Borgo E, Albisetti W (2010). Anterior cruciate ligament reconstruction with synthetic grafts. A review of literature. International Orthopaedics..

[73] Corella F, Ocampos M, Cerro MD (2018). Arthroscopic scaphoid 3D Test for scapholunate instability. Journal of Wrist Surgery..

[74] Mathoulin CL (2017). Indications, techniques, and outcomes of arthroscopic repair of scapholunate ligament and triangular fibrocartilage complex. Journal of Hand Surgery (European Volume).

[75] Mathoulin CL, Dauphin N, Wahegaonkar AL (2011). Arthroscopic dorsal capsuloligamentous repair in chronic scapholunate ligament tears. Hand Clinics.

[76] Corella F, Del Cerro M, Ocampos M, Simon de Blas C, Larrainzar-Garijo R (2017). Arthroscopic scapholunate ligament reconstruction, volar and dorsal reconstruction. Hand Clinics.

[77] Vekris MD, Mataliotakis GI, Beris AE (2011). The scapholunate interosseous ligament afferent proprioceptive pathway: A human in vivo experimental study. Journal of Hand Surgery (American Volume).

[78] Lindsay TAJ, Myers HR, Tham S (2021). Ligamentization and remnant integration: review and analysis of current evidence and implications for scapholunate reconstruction. Journal of Wrist Surgery..

[79] Watson HK, Ballet FL (1984). The SLAC wrist: Scapholunate advanced collapse pattern of degenerative arthritis. Journal of Hand Surgery (American Volume)..

[80] O'Meeghan CJ, Stuart W, Mamo V, Stanley JK, Trail IA (2003). The natural history of an untreated isolated scapholunate interosseus ligament injury. The Journal of Hand Surgery: British..

[81] Crisco JJ, Coburn JC, Moore DC, Akelman E, Weiss AP, Wolfe SW (2005). In vivo radiocarpal kinematics and the dart thrower's motion. Journal of Bone and Joint Surgery. American Volume.

[82] Anderson H, Hoy G (2016). Orthotic intervention incorporating the dart-thrower's motion as part of conservative management guidelines for treatment of scapholunate injury. Journal of Hand Therapy.

[83] Schwartz DA (2016). An alternative fabrication method of the dart thrower's motion orthosis (also known as the dart orthosis). Journal of Hand Therapy.

[84] Braidotti F, Atzei A, Fairplay T (2015). Dart-Splint: An innovative orthosis that can be integrated into a scapho-lunate and palmar midcarpal instability re-education protocol. Journal of Hand Therapy..

[85] Garcia-Elias M, Alomar Serrallach X, Monill SJ (2014). Dart-throwing motion in patients with scapholunate instability: A dynamic four-dimensional computed tomography study. Journal of Hand Surgery (European Volume).

[86] Garg R, Kraszewski AP, Stoecklein HH (2014). Wrist kinematic coupling and performance during functional tasks: Effects of constrained motion. Journal of Hand Surgery (American Volume)..

[87] Hagert E, Garcia-Elias M, Forsgren S, Ljung BO (2007). Immunohistochemical analysis of wrist ligament innervation in relation to their structural composition. Journal of Hand Surgery (American Volume)..

[88] Wolff AL, Wolfe SW (2016). Rehabilitation for scapholunate injury: Application of scientific and clinical evidence to practice. Journal of Hand Therapy.

[89] Esplugas M, Garcia-Elias M, Lluch A, Llusá PM (2016). Role of muscles in the stabilization of ligament-deficient wrists. Journal of Hand Therapy.

